# Evaluating the Impact of Abrupt Changes in Forest Policy and Management Practices on Landscape Dynamics: Analysis of a Landsat Image Time Series in the Atlantic Northern Forest

**DOI:** 10.1371/journal.pone.0130428

**Published:** 2015-06-24

**Authors:** Kasey R. Legaard, Steven A. Sader, Erin M. Simons-Legaard

**Affiliations:** School of Forest Resources, University of Maine, Orono, Maine, United States of America; Chinese Academy of Sciences, CHINA

## Abstract

Sustainable forest management is based on functional relationships between management actions, landscape conditions, and forest values. Changes in management practices make it fundamentally more difficult to study these relationships because the impacts of current practices are difficult to disentangle from the persistent influences of past practices. Within the Atlantic Northern Forest of Maine, U.S.A., forest policy and management practices changed abruptly in the early 1990s. During the 1970s-1980s, a severe insect outbreak stimulated salvage clearcutting of large contiguous tracts of spruce-fir forest. Following clearcut regulation in 1991, management practices shifted abruptly to near complete dependence on partial harvesting. Using a time series of Landsat satellite imagery (1973-2010) we assessed cumulative landscape change caused by these very different management regimes. We modeled predominant temporal patterns of harvesting and segmented a large study area into groups of landscape units with similar harvest histories. Time series of landscape composition and configuration metrics averaged within groups revealed differences in landscape dynamics caused by differences in management history. In some groups (24% of landscape units), salvage caused rapid loss and subdivision of intact mature forest. Persistent landscape change was created by large salvage clearcuts (often averaging > 100 ha) and conversion of spruce-fir to deciduous and mixed forest. In groups that were little affected by salvage (56% of landscape units), contemporary partial harvesting caused loss and subdivision of intact mature forest at even greater rates. Patch shape complexity and edge density reached high levels even where cumulative harvest area was relatively low. Contemporary practices introduced more numerous and much smaller patches of stand-replacing disturbance (typically averaging <15 ha) and a correspondingly large amount of edge. Management regimes impacted different areas to different degrees, producing different trajectories of landscape change that should be recognized when studying the impact of policy and management practices on forest ecology.

## Introduction

Forest policy and management practices within the U.S. have changed substantially following widespread dissatisfaction with management overly focused on the production of wood fiber and game species habitat. Over the past several decades, managers of public and private lands have to varying degrees incorporated a much wider set of objectives including the protection or provision of amenities, biodiversity, and ecosystem services [1,2]. Much of this change followed from recognition that management practices had undermined the landscape conditions needed to support certain forest values. Advances in scientific knowledge, stakeholder engagement, and government oversight of public interests have led to changes in public policy and private forest practices intended to improve the function of managed forest landscapes [1–3]. There are many, varied mechanisms of change. Management has evolved in response to public perception and market incentives. More abrupt changes have resulted from legislation and implementation of forest policy by government at all levels, from municipal to federal. State governments have been particularly active in legislating and enforcing regulatory programs [3,4]. Due to the complexity of ecological, economic, and social issues intertwined in the problem of forest management, regulatory programs are put into place with incomplete knowledge of future effects.

The sustainable management of forest landscapes and development of effective forest policy requires an understanding of the functional relationships between management practices, changes in landscape conditions, and ecological response. Abrupt changes in forest policy or other drivers of landscape dynamics make it fundamentally more difficult to evaluate these relationships. Because ecological processes operate over a wide range of temporal scales, responses to landscape change are time-dependent. Changes in species presence or abundance are frequently delayed following periods of rapid landscape change, and ecological communities take time to equilibrate to new landscape dynamics imposed by new management practices [5–8]. Delayed responses may effectively decouple ecological processes from recent patterns of landscape change [7]. The degree to which this occurs will vary depending on species life histories and the spatiotemporal dynamics of forest disturbance and recovery [7,8], but in general the ecological effects of forest policy change may emerge over long timeframes. This may be particularly true where past management practices imposed landscape conditions that persist for long periods. Legacies of past management practices (e.g., forest composition, spatial configuration of stand types) persist because they limit management options or alter patterns of natural disturbance or succession [9,10]. Unrecognized legacies and lagged responses may confound the attribution of observed ecological impacts to specific management practices.

Empirical studies of forest loss or fragmentation effects commonly rely on a space-for-time substitution [11], where replicate landscapes or patches are selected based on the current amount or configuration of forest (e.g., [12,13]). Although the intent is to study a fundamentally dynamic process, replication occurs in space rather than time, and landscape disturbance history is treated as an extraneous variable that is not controlled by experimental design. Inferences require the assumption that disturbance history acts as a random error term [14] when in fact it may be confounded with the experimental variables of current forest amount or configuration [8]. Studies that are intended to reveal impacts of landscape change should integrate disturbance history or temporal variability of landscape condition into study design (e.g., [15]). Similarly, where different management practices have been imposed at different times, knowledge of management history is needed to differentiate the consequences of contemporary practices from persistent impacts of past practices. Empirical evidence will otherwise be difficult to establish following abrupt changes in management regimes, when empirical study is perhaps most needed.

Satellite images provide the synoptic views needed to characterize forest conditions and landscape change. The ~40-year depth of the Landsat image archive in particular facilitates studies of forest landscape dynamics. However, there are relatively few retrospective analyses of landscape dynamics following abrupt changes in forest management practices. In the Pacific Northwest region of the U.S., Landsat image time series have been used to address the consequences of federal forest policy change (e.g., [16,17]). Landsat-derived forest cover maps and disturbance time series have been used to evaluate changes in forest conditions following the collapse of socialism in Eastern Europe and the former Soviet Union (e.g., [18,19]). In these cases, disturbance rates or measures of landscape change were summarized over time periods of interest (i.e., periods before and after policy change or sociopolitical reform) and over study areas defined by political boundaries, ecoregions, or image extents. Results provide summaries of change in useable forms, but the spatiotemporal dynamics of landscape change are resolved only in so far as they are partitioned by predetermined time periods or study areas. Empirical study of ecological processes affected by management requires knowledge of how management practices have influenced landscape dynamics across a range of ecologically relevant scales, but the spatial and temporal heterogeneity of management effects are difficult to synthesize over large areas and long time periods.

The Atlantic Northern Forest of the northeastern U.S. encompasses roughly 11 million hectares within a transition zone between the northern boreal forest and the southern temperate deciduous-dominant forest. A substantial portion of this area lies within northern Maine, the largest contiguous block of undeveloped forestland in the nation (~4 Mha). Despite a long history of logging and commercial management for fiber production, major changes in management practices within recent decades have led to contemporary landscape conditions with little historical precedent. The spruce-fir forests of the region are subject to periodic infestations of the eastern spruce budworm (*Choristoneura fumiferana* (Clem.)), a native pest that causes widespread defoliation and mortality of balsam fir (*Abies balsamea*) and spruce (*Picea spp*.) trees [20,21]. Maine's last outbreak occurred ca. 1972–1988 and stimulated broad-scale salvage harvesting by clearcut [20]. Public concern over the size of salvage clearcuts led to the passage of the Maine Forest Practices Act (FPA) [22] in 1989 and its implementation in 1991. The FPA fundamentally changed management practices by placing restrictions and disincentives on clearcutting. As a proportion of annual harvest area, clearcuts fell from 44% in 1989 to 10% in 1994 [23] and less than 5% by 2000 [24].

Management practices in Maine have elicited concerns regarding the sustainable provision of forest values. During the budworm outbreak, salvage logging rates were well above recognized long-term allowable levels [20]. Regeneration failures within salvage clearcuts resulted in the conversion of large areas of spruce-fir forest to deciduous and mixed types [21]. Following implementation of the FPA, state records indicate that annual harvest area roughly doubled during the 1990s [23,24] as landowners maintained similar extraction rates via partial harvest practices that require a larger footprint to achieve the same volume removal. The spatial dynamics associated with implementation of the FPA have been partially assessed. Analysis of a Landsat-derived disturbance time series (1988–1999) found that implementation of the FPA coincided with a change toward fewer and smaller clearcut patches, and fewer but larger partial harvest patches [25]. A subsequent analysis of Landsat-derived forest cover and disturbance data found that harvest patches of the 1980s were larger and more compact than patches of the 1990s, but this study did not differentiate clearcuts from partial harvests [26]. Management practices and harvest rates differ between private forestland owners [27,28], suggesting important differences in post-FPA landscape change. However, rates and patterns of landscape change attributable to pre- and post-FPA management regimes have not been sufficiently resolved to support a more complete assessment of policy impact on landscape dynamics.

The objective of our research was to characterize predominant patterns of cumulative landscape change in the Atlantic Forest of northern Maine, and to evaluate how pre- and post-FPA management regimes have influenced landscape conditions across space and time. We used Landsat imagery and forest inventory data to develop and validate forest composition maps and a time series of forest harvest maps (1973–2010). We modeled predominant temporal patterns of harvesting and segmented a large study area into groups of landscape units with similar harvest histories. We then linked harvest history with changes in landscape composition and configuration in order to characterize the evolution of landscape conditions in response to forest management practices before and after abrupt change induced by the FPA. Our approach provided an objective synthesis of predominant patterns of change associated with specific landscape units, with the spatial and temporal resolution needed to attribute change to different management regimes.

## Methods

### Study Area

**Fig 1:**
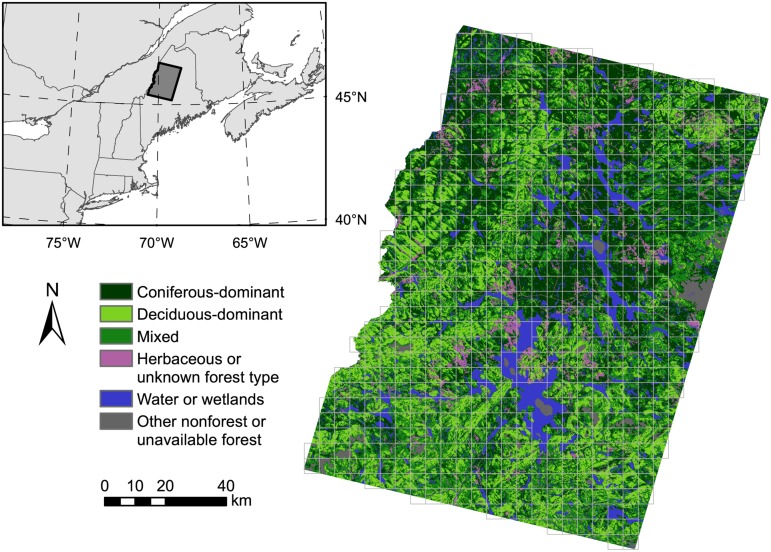
Study area. Northern Maine, U.S.A. study area with 5 km square sample landscape units superimposed. Harvesting trends and patterns of landscape change were calculated for forestland assumed available for harvest. Mapped forest composition classes demonstrate the spatial distribution of general forest types at the onset of our study period (1975). State and provincial boundaries displayed in the inset map were obtained from the National Atlas of the U.S. (Political Boundaries) and the Atlas of Canada (National Frameworks Data, Census Subdivisions and Population Ecumene).

Our northern Maine, U.S.A. study region (Fig 1) was defined by the overlap of Landsat images and includes ~1.5 Mha of forestland. Rural development and agriculture are concentrated in a few small areas. Topography is generally flat or rolling with occasional low mountains and an extensive network of rivers, lakes, and wetlands. Forest types are typical of the Atlantic Northern Forest and generally occur in predictable patterns associated with climatic gradients and soil conditions determined by glacial deposition [29]. Northern hardwood species (*Acer rubrum*, *Acer saccharum*, *Betula alleghaniensis*, *Betula papyrifera*, *Fagus grandifolia*) predominate across lower hilltops and at mid-slope. Spruce-fir species (*Abies balsamea*, *Picea glauca*, *Picea mariana*, *Picea rubens*) predominate where soil or microclimatic conditions exclude the more demanding hardwoods. Mixedwood stands commonly occur along ecotones or as a result of successional dynamics following disturbance. Shade-intolerant hardwood species (e.g., *Populus tremuloides*, *Betula papyrifera*) are commonly found following intense disturbance. Periodic defoliation by spruce budworm is the most prominent form of natural disturbance. Windthrow is common but generally results in small canopy gaps [30]. Virtually all forestland is considered commercially productive [29] and roughly 90% is private. Public lands are interspersed and primarily state-owned.

### Data production

Forest harvest and composition maps were assembled from a time series of Landsat Multispectral Scanner (MSS), Thematic Mapper (TM), and Enhanced Thematic Mapper Plus (ETM+) images acquired during summer leaf-on conditions (Table 1). Consecutive images were spaced 1–4 years apart, as determined by the availability of high quality, predominantly cloud-free imagery. Mapping procedures were applied to forested pixels as identified by the 1993 Maine Gap Analysis Program (GAP) land cover map. The GAP map represents conditions near the midpoint of our time series, and discriminated forest from non-forest with an estimated 100% accuracy within our study area [31]. Not all forestland within our study area is operable or available for harvesting. To normalize harvest rates and metrics of landscape change according to the amount of available forestland in different landscape units, we masked forest pixels over 823 m (2700 ft) in elevation or >40% slope, as determined from the 1 arc-second National Elevation Dataset. Harvesting under these conditions has historically been allowed by special permit only, and we consider these areas inoperable or otherwise unavailable for harvest. Forested islands were masked as well, with the exception of one large island with a history of harvesting. Less than 3% of forestland was masked as unavailable. Refer to S1 Appendix for a detailed description of image processing performed prior to forest harvest and composition mapping.

**Table 1:** Landsat images used to map forest harvesting (1973–2010) and forest composition (1975 and 2004).

Acquisition date	Landsat sensor	Landsat satellite	% forestland under cloud/shadow
2010, August 30	TM	5	<0.1
2007, June 17^a^	TM	5	0.8
2004, June 10	TM	5	0.8
2001, May 25	ETM+	7	< 0.1
2000, August 26	ETM+	7	1.6
1999, June 13	TM	5	< 0.1
1997, June 23	TM	5	16.0
1995, July 4	TM	5	5.0
1993, September 16^b^	TM	5	15.9
1991, June 7^b^	TM	5	< 0.1
1988, September 2	TM	5	0.9
1988, September 2	MSS	5	0.9
1985, June 22^c^	MSS	5	4.3
1982, July 30	MSS	3	0.9
1978, August 11	MSS	2	< 0.1
1975, August 9	MSS	2	0
1973, July 23^c^	MSS	1	0.1

Images were acquired over Landsat Woldwide Reference System (WRS)-2 path 12, row 28 (1985–2010) and WRS-1 path 13, row 28 (1973–1982). Unless otherwise indicated, images were obtained from the U.S. Geological Survey Earth Resources Observation and Science Center.

^a^Areas of cloud cover filled with TM image data acquired on 22 August 2007.

^b^Available through the Maine GAP Analysis Project.

^c^Available through the North American Landscape Characterization project.

#### Forest harvest mapping, 1973–2010

Forest harvest maps were produced using a change detection procedure based on vegetation index values calculated from sequential Landsat images. As initially described by Sader and Winne [32], forest canopy disturbance and recovery can be visualized using a three-band color composite image incorporating values of the normalized difference vegetation index (NDVI = [near-infrared - red] / [near-infrared + red]) acquired on three separate dates. Classification of the three-date NDVI data produces a thematic map depicting forest canopy changes [25]. Other vegetation indices may be substituted for the NDVI and the normalized difference moisture index (NDMI = [near-infrared - mid-infrared] / [near-infrared + mid-infrared]) has been found particularly effective in discriminating partial canopy disturbance using TM/ETM+ data [33,34]. Whereas the NDVI represents a normalized contrast between near-infrared and red reflectance, the NDMI contrasts near-infrared and mid-infrared reflectance.

The improved sensitivity of the NDMI to partial canopy disturbance is generally attributable to the heightened sensitivity of mid-infrared wavelengths to differences in forest canopy structure, leaf area, and biomass [34,35].

We classified three-date NDMI and NDVI composites to produce forest change maps from TM/ETM+ and MSS image sequences, respectively. MSS imagery lacks a mid-infrared band required for calculation of the NDMI. This difference, coupled with reduced spatial and radiometric resolution, limits the efficacy of MSS imagery for detection of partial canopy disturbance. Disturbances mapped using MSS imagery (1973–1988) represent stand-replacing events, predominantly spruce budworm salvage clearcuts. Disturbances mapped using TM/ETM+ imagery (1988–2010) represent a wide range of intensities, and we differentiated two intensity classes interpreted as stand-replacing and partial canopy disturbance. The stand-replacing class was intended to represent harvests in which a new cohort was established following removal of a large proportion of the canopy, whether by clearcut as defined by the FPA [36,37] or by other harvest types. Mapped disturbance events were almost exclusively the result of harvest operations and we therefore refer to our data as a time series of forest harvest maps.

Harvest maps were produced by unsupervised classification of overlapping three-date NDVI or NDMI image sequences (e.g., 1973-1975-1978, 1975-1978-1982, …). Classification of a three-date sequence mitigates the impact of cloud cover in the second image provided the first and third give clear views. An ISODATA algorithm applied to each three-date composite produced 50 statistical classes that were interpreted into forest disturbance, regrowth, and no-change information classes. Stand-replacing and partial harvest classes derived from TM/ETM+ imagery were differentiated based on the relative magnitude of NDMI change, guided by visual interpretation of Landsat imagery and available aerial photography. Confusion between light partial harvests and changes induced by factors such as atmospheric effects or interannual variability in forest phenology were resolved through on-screen editing [38]. Individual harvest maps were compiled for each time interval (e.g. 1973–1975, 1975–1978, …) by combining equivalent harvest classes from overlapping three-date change maps. Harvest patches less than 0.81 ha in size were removed, and a 3x3 pixel majority filter was applied to consolidate patch boundaries and simplify the patch structure of maps produced from TM/ETM+ imagery to more closely match maps produced from the lower resolution MSS imagery.

We produced a time series of maps depicting cumulative harvest impact (1975–2010) by overlaying successive harvest maps. For each time series date, a pixel was labeled as regenerating forest if preceding intervals included a harvest 1973–1988 or a stand-replacing harvest 1988–2010. A pixel was labeled as partially harvested if preceding intervals included only a single partial harvest. When preceding intervals included multiple partial harvests, pixels were labeled as regenerating forest, reflecting the anticipated ecological and silvicultural effects of multiple entries within the ~20-year period over which partial harvests were mapped (1988–2010). For each date of our time series, the result depicts the cumulative footprint of harvest operations since 1973.

#### Forest type mapping, 1975 and 2004

We mapped forest composition using equivalent unsupervised classification methods applied to each of the 1975 MSS and 2004 TM images. Dates were selected on the basis of cloud cover and image quality. For the purpose of forest type mapping, small areas of cloud cover in the 2004 image were replaced with data from the 2001 ETM+ image. Statistical classes produced from an ISODATA algorithm were aggregated to coniferous-dominant (>75% coniferous), deciduous-dominant (>75% deciduous), and mixed type classes through visual interpretation of Landsat imagery, with reference to available aerial photography and existing land cover maps. In some previously disturbed areas, exposed soils, woody debris, or herbaceous vegetation precluded the assignment of forest type and pixels were instead assigned to an indeterminate class. Patches less than 0.81 ha in size were removed and a 3x3 majority filter was applied to each map to consolidate patch boundaries and simplify the 2004 patch structure to more closely match the 1975 data.

Assignment of ISODATA classes to forest types was subjective and sometimes difficult. A mistaken assignment could lead to bias in the representation of forest type extent. If for example pixels representing forest with a deciduous component of 70–75% were mistakenly committed to the deciduous-dominant class rather than the mixed class, the extent of the deciduous class would be overestimated according to the class definition of >75% deciduous. We used validation data obtained from field plots (described below and in S2 Appendix) to iteratively refine the aggregation and labeling of ISODATA classes to ensure that the 1975 and 2004 maps provide unbiased representations of forest type classes at the same thresholds of forest composition. For each map, we identified coniferous- and deciduous-dominant class thresholds for which omission and commission errors were balanced. To do so, we varied coniferous and deciduous threshold values from 50–95% in increments of 5%, assigned reference class labels based on threshold values, and calculated omission and commission error rates. We iteratively refined the maps and reevaluated error rates until a reasonable balance was achieved at the same threshold for both maps, facilitating meaningful comparisons of class extent between maps.

### Data validation

The U.S. Forest Service Forest Inventory and Analysis (FIA) Program provides quality-assured measurements of forest attributes from a national network of field plots adhering to a systematic sampling design [39]. We made extensive use of FIA data as a statistically rigorous basis for map validation. However, use of field plot data for map validation is subject to uncertainty arising primarily from mismatches in location and scale between field plots and map pixels. Validation using FIA data should be considered an assessment of agreement with an accepted and widely utilized source of information on forest conditions, rather than an assessment of accuracy against ground truth. Here we provide an overview of our validation procedures; details are provided in S2 Appendix.

#### Harvest time series validation

FIA estimates of forest age have been used to validate Landsat-derived disturbance time series under the assumption that trees sampled for age estimation germinated at the time of disturbance [40]. However, age is an imprecise estimate of the timing of past disturbance due to estimation uncertainty and variation in the timing of germination with respect to canopy removal. A new cohort may have been established from a seed source several years following disturbance or as advance regeneration prior to disturbance. Alternatively, visual interpretation of Landsat imagery is a credible means of dating disturbance events [25,38,41]. Unfortunately, visual discrimination of harvest intensity at the pixel scale is highly subjective. We developed a validation procedure based on visual interpretation of Landsat imagery over FIA plot locations. Image interpretation was used to date harvest events; FIA plot data were used to discriminate stand-replacing and partial harvests.

In Maine, the contemporary FIA inventory design was established in 1999, with 20% of plots surveyed annually during sequential 5-year cycles. Although data are available from plots measured during earlier inventories, coordinate locations are known for only a fraction of those plots. We therefore used data collected during contemporary inventory cycles to discriminate past harvest intensity. A harvest event identified by image interpretation was labeled stand-replacing provided FIA age dated stand origin to 1970 or later (allowing for advance regeneration prior to 1973) and field crews labeled the stand as either sapling or poletimber. However, for plots harvested after 1999, recorded stand age was an unreliable indicator of stand-replacing disturbance because age estimates frequently corresponded to a few residual stems rather than a newly established cohort. In these cases, intensity classes were discriminated using plot measurements made during consecutive 5-year inventory cycles; a harvest was labeled stand-replacing if plot basal area (cross-sectional area of stems measured at 1.37 m) was reduced by >70%.

Our validation sample of 509 plots was insufficient to produce reasonably precise accuracy estimates for individual time series intervals. We therefore aggregated intervals into six validation classes: 1973–1988 stand-replacing harvest, 1988–1999 stand-replacing harvest, 1988–1999 partial harvest, 1999–2010 stand-replacing harvest, 1999–2010 partial harvest, and intact mature forest (no history of harvest, 1973–2010). Map and reference validation class labels were assigned in a manner consistent with the construction of cumulative harvest maps. Where multiple entries occurred, labels were assigned based on the date of first stand-replacing disturbance. Where multiple partial harvests occurred, labels of either 1988–1999 or 1999–2010 stand-replacing were assigned based on the date of second entry. Map and reference labels were compiled into an error matrix. Overall accuracy, user accuracy (the complement of class commission error), producer accuracy (the complement of class omission error), and corresponding standard error estimates were calculated by poststratification [42,43]. Additionally, we evaluated the accuracy of our 2010 cumulative harvest map by further aggregating validation classes into regenerating, partially harvested, and intact mature forest.

#### Forest type validation

The 1975 and 2004 forest type maps were validated using FIA plot measurements of coniferous and deciduous live tree basal area collected during 1980–1982 and 1999–2003 inventories, respectively. Differences in dates between maps and field inventories were resolved by excluding samples where intervening harvests occurred. For 2004 map validation, we excluded plots mapped as harvested from 1999–2004; for 1975, we excluded plots mapped as harvested from 1975–1982. A sample of 445 plots remained for validation of the 2004 map; only 70 plots were available for validation of the 1975 map. As previously described, we identified coniferous-dominant and deciduous-dominant class thresholds for which errors were best balanced and mapped class extents least biased. Following refinements made to improve consistency between maps, an error matrix was compiled for each map based on selected threshold values. Estimates of overall, user, and producer accuracy were calculated by poststratification [42,43].

### Data analysis

To quantify harvest rates through time, identify trends, and associate trends with changes in landscape conditions, we tessellated our study area into landscape units using a 5 km square grid (Fig 1). A 5 km grid cell size was a somewhat arbitrary compromise: small enough to resolve spatial variations in harvest history and consequent landscape change, but large enough to calculate meaningful trends in harvest rates and landscape pattern metrics. We excluded grid cells with <50% available forest or <5% of available forest harvested from 1975–2010 (17 cells). A sample of 608 grid cells remained.

#### Empirical orthogonal function (EOF) analysis of cumulative harvest time series

An EOF analysis identifies a sequence of uncorrelated patterns or modes of variability that characterize variation within a two-dimensional data set [44]. EOF analysis is commonly employed in meteorology and oceanography, where conventional applications decompose time series of geospatial data into characteristic spatial patterns whose contributions to observed variation change through time. EOF outcomes can just as readily be interpreted as characteristic temporal patterns whose contributions to observed variation differ between locations (e.g., [45]). We performed an EOF analysis to identify characteristic temporal patterns of variation in cumulative harvest area sampled across our 5 km grid. Cumulative harvest time series were arranged as rows within a matrix X (M = 608 rows; N = 15 columns). EOF analysis decomposed X into matrices A and B such that X = A·B (A is MxN; B is NxN). The rows of B represented a sequence of mutually uncorrelated patterns of temporal variability referred to as empirical orthogonal functions (EOFs). The columns of A represented a complementary set of spatial patterns referred to as amplitude functions. The observed cumulative harvest time series were thereby represented as linear combinations of temporal EOFs, whose contributions were given by the spatial amplitude functions. EOF analysis is mathematically equivalent to principal component analysis (PCA). The temporal EOFs are computed as the eigenvectors of the dispersion matrix X^T^X and are equivalent to the loading vectors or principal components of a PCA. The spatial amplitudes correspond to the PCA scores obtained by projecting the time series of X onto the subspace spanned by the EOFs.

A traditional EOF analysis or PCA is sensitive to extreme observations and outliers, which can distort the outcome such that dominant modes of variability represent contrasts between anomalous and regular observations rather than patterns of variability within regular observations [46]. We performed our EOF analysis using a variant of the robust algorithm ROBPCA that is also suitable for skewed distributions [47,48]. Cumulative harvest area distributions were significantly skewed for 9 of the 15 time series dates (medcouple, p < 0.05; [49]). The ROBPCA algorithm is based on robust estimation of the covariance matrix from a specified proportion of samples with minimal outlyingness. The proportion of samples used may range from 0.5 to 1, and the value selected represents a compromise between the robustness and efficiency of the estimate. We used a sample proportion of 0.9 following exploratory analysis which suggested that relatively few outliers were present. Outcomes were not sensitive to the exact value used. Prior to analysis, we centered and scaled the cumulative harvest time series by removing the median and dividing by the median absolute deviation (computed across all cells, for each observation date). Scaling improved the fit of the EOF model for intervals near the beginning and end of the study period.

Paired EOFs and amplitude functions comprise orthogonal modes of variability, ordered by the amount of total variance explained. We modeled cumulative harvest time series as linear combinations of dominant EOFs, selected based on the proportion of overall variance explained by successive modes. By including only dominant modes, modeled time series represent statistically coherent variability in harvesting patterns that occurred over large portions of the study area. The ROBPCA algorithm provides a measure of orthogonal distance between samples and the subspace defined by dominant EOF modes. Unusually large orthogonal distances indicate outlying samples that do not conform to characteristic patterns defined by dominant modes. We identified 12 orthogonal outliers using the ROBPCA nominal cutoff value [47] and excluded them from subsequent analyses.

#### Predominant patterns of harvesting and landscape change

To classify predominant temporal patterns of harvesting from the EOF analysis and to associate those patterns with groups of grid cells, we performed an agglomerative hierarchical clustering [50] of modeled time series. Using Ward's minimum variance method [51], we produced a dendrogram and identified clusters of grid cells with similar harvest history. The mean of the modeled time series from each cluster demonstrated a predominant pattern of harvesting through time, representative of a group of landscape units.

Landscape composition metrics were calculated for grid cells and averaged within groups to evaluate changes associated with predominant harvesting trends. Within our time series of cumulative harvest maps, available forestland was classified as either regenerating, partially harvested, or intact mature forest (no harvesting, 1973–2010). The EOF and cluster analyses produced time series of cumulative harvest area, the reciprocal of intact mature forest area. We also produced time series of cumulative partial harvest and regenerating forest area to evaluate changes in harvest intensity associated with predominant harvesting trends. Available forestland was summarized by 1975 forest type to associate harvest history and landscape change with initial landscape composition. To evaluate composition change as a legacy of harvest practices, we quantified forest type change between 1975 and 2004 for areas harvested before 2004. Composition change in unharvested forestland was not evaluated as part of this research.

Early successional and intact late successional forest patches are landscape elements of particular interest, given the expansion of partial harvest practices in the 1990s. Cumulative changes in the patch configuration of regenerating and intact mature forest were evaluated by calculating time series of landscape metrics. We selected a small number of metrics of general relevance to forest ecology that quantify primary aspects of class configuration thought to have been affected by changes in management practices. Metrics were calculated using Fragstats version 4.2 [52]. Area-weighted mean patch size (Area_AM; ha) and area-weighted mean fractal dimension (Frac_AM; unitless) were calculated as measures of average patch area and shape complexity. Patch density (PD; patches/100 ha) was calculated as a simple measure of patch subdivision. The prevalence of edge conditions was quantified by edge density (ED; m/ha). An eight-neighbor rule was used for patch delineation. Non-forest and unavailable forest classes were treated as external to the landscape in order to normalize metric values across grid cells containing different amounts of managed forestland. Grid cell borders and non-forest edges were not included in the calculation of ED. Unavailable forest edges were included in the calculation of regeneration ED but not intact mature ED (i.e., unavailable forest was treated as intact mature forest for the purpose of calculating ED).

## Results

### Data validation

Harvest validation classes were mapped with an overall accuracy of 88% (Table 2). User and producer accuracies for the intact mature class were high (>95%) and well balanced, indicating an accurate depiction of overall harvest footprint. Stand-replacing harvests of 1973–1988 were mapped with high accuracy (89–91%) compared to subsequent periods in which confusion between stand-replacing and partial harvests reduced accuracies for both classes (75–91%). Individual class accuracies were reasonably well balanced save for 1988–1999 stand-replacing harvests, which may have been systematically under-represented. However, the criteria used to establish reference class labels differed between periods, and differences in class accuracy estimates may partly reflect inconsistency in discriminating harvest intensity from available validation data. The 2010 cumulative harvest map depicted regenerating and partially harvested forest with >86% and >75% accuracy, respectively (Table 3). Overall accuracy associated with regenerating, partially harvested, and intact mature forest classes approached 90%.

**Table 2:** Error matrix and accuracy estimates for validation classes aggregated from the 1973–2010 forest harvest time series.

	Reference validation class							
Mapped validation class	1973–1988 stand-replacing	1988–1999 stand-replacing	1988–1999 partial harvest	1999–2010 stand-replacing	1999–2010 partial harvest	Intact mature^a^	Total	User accuracy
1973–1988 stand-replacing	73	1	2	0	0	4	80	91.3% (3.2%)
1988–1999 stand-replacing	1	49	3	1	0	0	54	90.7% (4.0%)
1988–1999 partial harvest	3	7	36	0	0	2	48	75.0% (6.3%)
1999–2010 stand-replacing	1	1	1	30	7	0	40	75.0% (6.9%)
1999–2010 partial harvest	1	0	0	7	33	3	44	75.0% (6.6%)
Intact mature^a^	4	2	2	0	3	232	243	95.5% (1.3%)
Total	83	60	44	38	43	241	509	
Producer Accuracy^b^	88.6% (2.7%)	77.2% (4.4%)	83.9% (5.0%)	81.6% (5.0%)	76.4% (5.1%)	95.3% (1.5%)		
Overall Accuracy^b^	87.9% (1.4%)							

Standard error estimates are provided in parentheses.

^a^No harvesting, 1973–2010.

^b^Estimated by poststratification using known pixel counts.

**Table 3:** Error matrix and accuracy estimates for the 2010 cumulative harvest map.

	Reference forest class				
Mapped forest class	Regenerating	Partially harvested	Intact mature^a^	Total	User accuracy
Regenerating	157	13	4	174	90.2% (2.3%)
Partially harvested	18	69	5	92	75.0% (4.5%)
Intact mature^a^	6	5	232	243	95.5% (1.3%)
Total	181	87	241	509	
Producer accuracy^b^	86.6% (2.0%)	81.3% (3.6%)	95.4% (1.5%)		
Overall accuracy^b^	89.3% (1.3%)				

Standard error estimates are provided in parentheses.

^a^No harvesting, 1973–2010.

^b^Estimated by poststratification using known pixel counts.

Forest type classes for 1975 and 2004 were mapped with overall accuracies of 76% and 68%, respectively (Tables 4 and 5). Individual class accuracy estimates were similarly lower for 2004 than for 1975, presumably due to more heterogeneous landscape conditions. Off-diagonal entries in error matrices indicated confusion between the mixed class and both coniferous- and deciduous-dominant classes. There was little confusion between coniferous and deciduous classes. Error matrices and accuracy estimates were derived using class definitions for which omission and commission errors were best balanced and class accuracies acceptably high for both maps. Using coniferous-dominant and deciduous-dominant class thresholds of >80% and >70% basal area, respectively, errors were very well balanced for 2004 forest type classes (Table 5). User and producer accuracies for the 1975 map (Table 4) suggested under-representation of coniferous forest area and over-representation of mixed forest under these same class definitions, but the relatively small validation sample and correspondingly large standard error estimates made this inconclusive. Available validation data suggested that user and producer accuracies were best balanced under these class definitions.

**Table 4:** Error matrix and accuracy estimates for the 1975 forest type map.

	Reference forest type				
1975 mapped forest type	Coniferous^a^	Mixed	Deciduous^b^	Total	User accuracy
Coniferous^a^	15	3	0	18	83.3% (9.0%)
Mixed	6	22	4	32	68.8% (8.3%)
Deciduous^b^	1	3	16	20	80.0% (9.2%)
Total	22	28	20	70	
Producer accuracy^c^	74.0% (6.6%)	76.5% (6.4%)	78.9% (8.5%)		
Overall accuracy^c^	76.2% (5.0%)				

Standard error estimates are provided in parentheses.

^a^>80% coniferous basal area.

^b^>70% deciduous basal area.

^c^Estimated by poststratification using known pixel counts.

**Table 5:** Error matrix and accuracy estimates for the 2004 forest type map.

	Reference forest type				
2004 mapped forest type	Coniferous^a^	Mixed	Deciduous^b^	Total	User accuracy
Coniferous^a^	107	45	1	153	69.9% (3.7%)
Mixed	43	122	36	201	60.7% (3.5%)
Deciduous^b^	6	31	118	155	76.1% (3.4%)
Total	156	198	155	509	
Producer accuracy^c^	70.7% (2.9%)	59.9% (2.7%)	76.5% (2.9%)		
Overall accuracy^c^	68.3% (2.0%)				

Standard error estimates are provided in parentheses.

^a^>80% coniferous basal area.

^b^>70% deciduous basal area.

^c^Estimated by poststratification using known pixel counts for mapped forest type classes.

### EOF analysis of cumulative harvest time series

**Fig 2:**
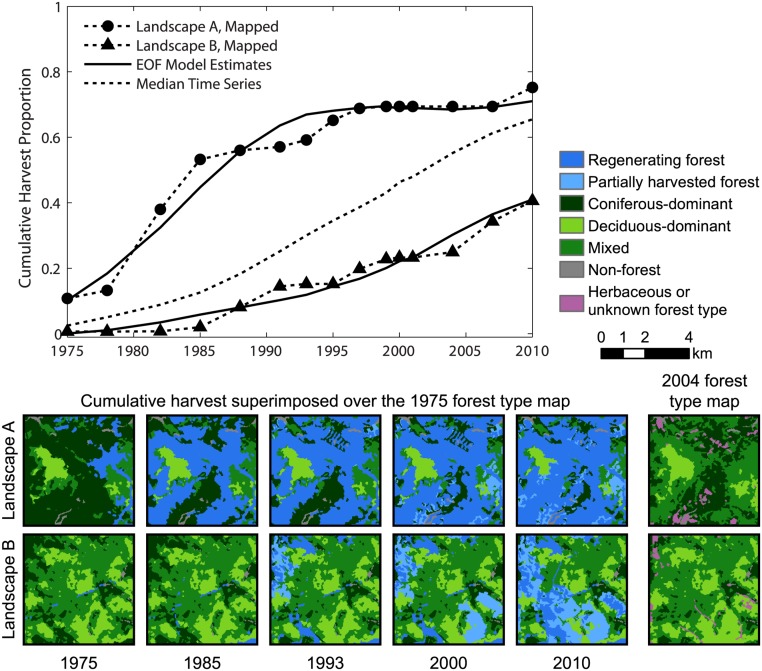
Forest harvest trends and landscape change for two sample grid cells. Mapped and modeled cumulative harvest time series for two arbitrary sample grid cells, expressed as a proportion of available forestland. The median cumulative harvest time series (n = 608) is shown for reference. Images of landscape conditions include cumulative harvest impact superimposed over the 1975 forest type map for a subset of time series dates, and the 2004 forest type map. Comparison of the 1975 and 2004 forest type data indicates areas where intervening harvests induced changes in forest type.

By 2010, 61% of available forestland was mapped as harvested, and 40% regenerated by stand-replacing or multiple partial harvests. Averaged across all grid cells, harvest rates increased ca. 1985 and then remained quite constant at about 2% per year (median cumulative harvest time series; Fig 2). The EOF analysis decomposed cumulative harvest time series into characteristic patterns of deviation from the median series. We retained 3 dominant EOF modes, which collectively explained 92% of total variance of the centered and standardized time series (62%, 23%, and 7% of total variance). This 3-mode EOF model provided an adequate representation of temporal trends for the great majority of individual time series (>90% of variance captured at 78% of grid cells; <70% of variance captured at 2% of cells).

Time series of mapped and modeled cumulative harvest area at two sample locations (Fig 2) illustrate the suitability of the EOF model for representing trends and smoothing the irregularities resulting from more erratic year-to-year changes in harvest rates. For the first of these sample landscapes (Landscape A; Fig 2), harvest area increased rapidly through the first half of the study period (compared to the median time series), and then changed very little during the second half. The extensive harvesting of the 1970s and 1980s was predominantly stand-replacing and directed at coniferous forest. By 1985, more than half of available forestland was regenerated. As indicated by the 2004 forest type map, much of that area was converted from coniferous-dominant to mixed. Within the second sample landscape (Landscape B; Fig 2), harvesting consisted of both stand-replacing and partial canopy removals primarily during the second half of the study period within deciduous and mixed forest. Little harvesting occurred prior to 1985. Harvest rates were relatively modest between 1985 and 2004 and were somewhat elevated thereafter.

### Harvesting trends

**Fig 3:**
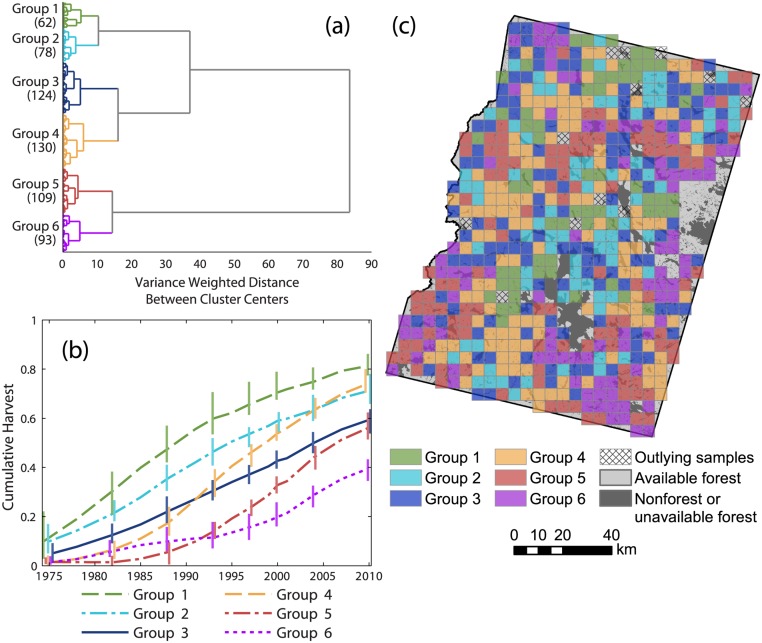
Predominant patterns of harvesting. (a) Dendrogram produced by agglomerative hierarchical clustering of modeled cumulative harvest time series. Six groups of landscape units were identified for subsequent analysis (sample sizes provided in parentheses). (b) Mean cumulative harvest area time series for each of the groups identified in (a), expressed as a proportion of available forestland. Vertical bars represent the interquartile range. Bars are provided at a subset of dates and are offset horizontally to improve visual clarity. (c) Spatial distribution of groups identified in (a). Hatching indicates outlying samples excluded from further analysis.

From hierarchical clustering of modeled cumulative harvest time series, we identified six well-defined groups (Fig 3a) ranging in size from 10% to 22% of grid cells. The mean time series from each group represented a predominant pattern of harvesting through time (Fig 3b). For groups 1–2 (24% of grid cells), harvest rates exceeded median rates during the first half of the study period, particularly for group 1, and then dropped during the second half. The group 3 time series closely resembled the median time series. Groups 4–6 (56% of grid cells) shared the characteristics of relatively little harvesting early on followed by elevated rates during later years. Group 5 was notable in that harvest rates were exceptionally low through the 1980s but very high through the 1990s and 2000s.

**Fig 4:**
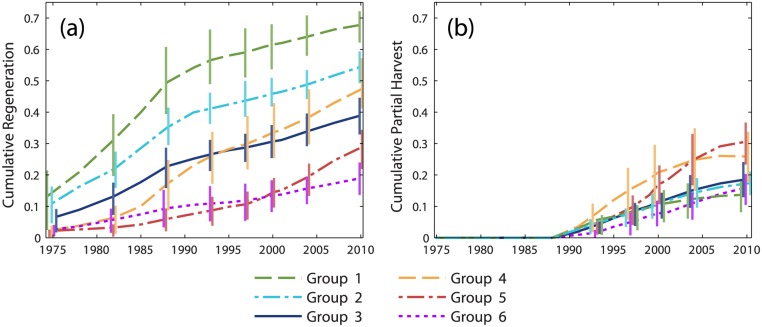
Time series of regenerating and partially harvested forest area. Cumulative time series of (a) regenerating forest area and (b) partially harvested forest area, expressed as a proportion of available forestland and averaged within groups identified by cluster analysis of modeled cumulative harvest time series. Vertical bars represent the interquartile range. Bars are provided at a subset of dates and are offset horizontally to improve visual clarity.

Time series of cumulative regenerating and partially harvested forest area (Fig 4) differ substantially between groups. Note that partial harvests were not mapped prior to 1988. Groups 1–2 were notable for rapid, heavy harvesting during the first half of the study period, followed by moderated rates of both stand-replacing and partial harvesting through the second half. Groups 4 and 5 were most strongly differentiated from other groups by high rates of partial harvesting, although high harvest rates during the 1990s and 2000s were sustained by both stand-replacing and partial harvests. Group 6 stand-replacing and partial harvest rates were low or moderate throughout the study period.

### Patterns of landscape change

**Fig 5:**
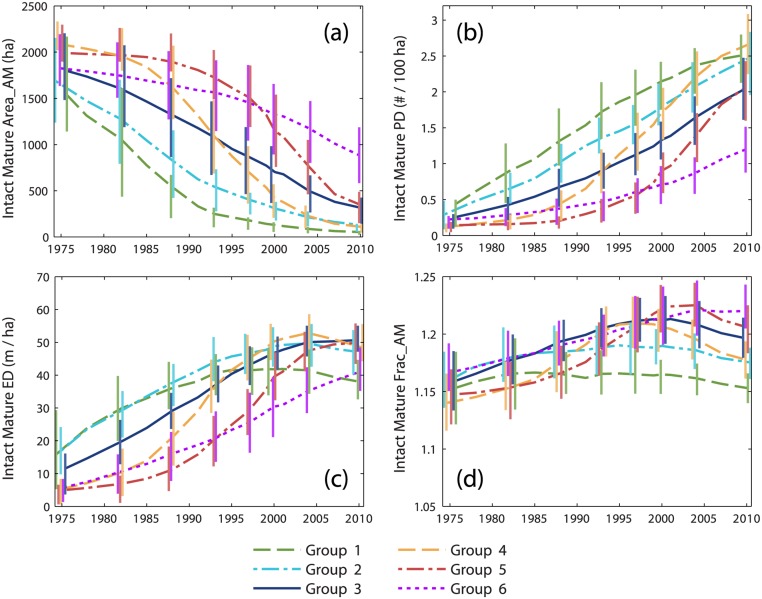
Time series of intact mature forest configuration metrics. Time series of cumulative change in (a) area-weighted mean patch size, (b) patch density, (c) edge density, and (d) area-weighted mean fractal dimension for intact mature forest, averaged within groups identified by cluster analysis of modeled cumulative harvest time series. Vertical bars represent the interquartile range. Bars are provided at a subset of dates and are offset horizontally to improve visual clarity.

Time series of landscape metrics quantified the cumulative effects of harvesting on forest configuration. Time series of average patch size for intact mature forest (Fig 5a) reflected trends in cumulative harvest area (Fig 3b); periods of rapid patch size reduction coincided with periods of rapid harvesting. Patch density (Fig 5b) increased through time, most rapidly in groups 4 and 5 during the 1990s and 2000s. For groups 1–5, the amount of edge between intact mature forest and harvested forest (Fig 5c) increased and then peaked as harvest area approached and then surpassed 50% of available forestland. The increase in edge density was most rapid in groups 4 and 5 during the 1990s. Trajectories of average patch shape complexity (Fig 5d) were similar in general character to those of edge density, but with peak values occurring somewhat earlier and with little change in groups 1–2 over the course of our study period.

**Fig 6:**
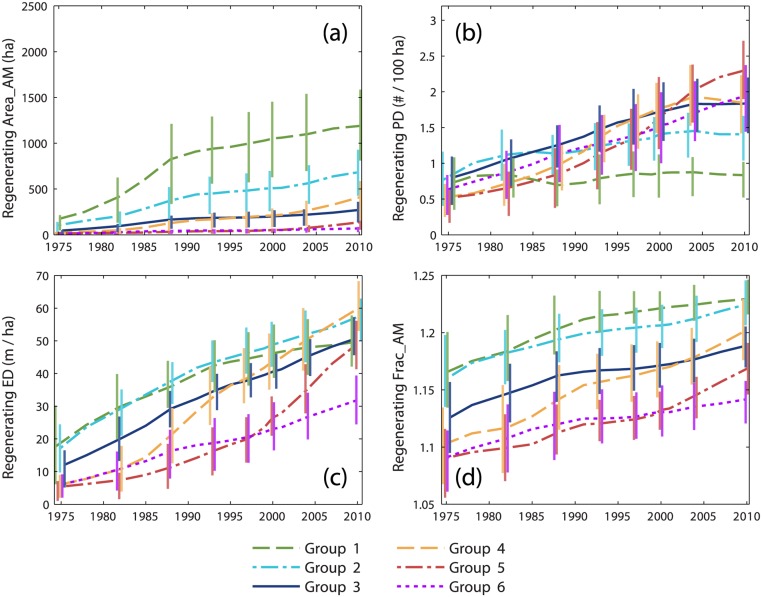
Time series of regenerating forest configuration metrics. Time series of cumulative change in (a) area-weighted mean patch size, (b) patch density, (c) edge density, and (d) area-weighted mean fractal dimension for regenerating forest, averaged within groups identified by cluster analysis of modeled cumulative harvest time series. Vertical bars represent the interquartile range. Bars are provided at a subset of dates and are offset horizontally to improve visual clarity.

Large differences between groups in time series of regenerating forest area (Fig 4a) were only partly reflected in configuration metrics. Changes in average regenerating forest patch size (Fig 6a) were greatest for group 1, increasing from less than 200 ha in 1975 to more than 800 ha by 1988. In contrast, average patch size remained low for groups 3–6. Group 5 values remained well below 200 ha despite relatively rapid increases in regenerating forest area during the 2000s (Fig 4a). Regenerating patch density (Fig 6b) generally increased throughout the study period, but this trend was less pronounced for group 2 and not apparent for group 1. The largest values of patch density were attained by group 5 following rapid increase during the 1990s and 2000s. Despite low rates of stand-replacing disturbance in group 6 (Fig 4a), patch density increased steadily and was quite high by 2010. The amount of regenerating forest edge (Fig 6c) was greatest for groups 1–2 until the 2000s when group 4 attained comparable levels following rapid gains beginning in the late 1980s. Group 5 edge density increased rapidly during the 2000s. Average patch shape complexity of regenerating forest (Fig 6d) increased during the first half of the study period, generally leveled somewhat during the 1990s, and then increased once more during the 2000s, most markedly for groups 4 and 5. Throughout the study period, regenerating patch shape complexity was greatest for groups 1–2.

**Fig 7:**
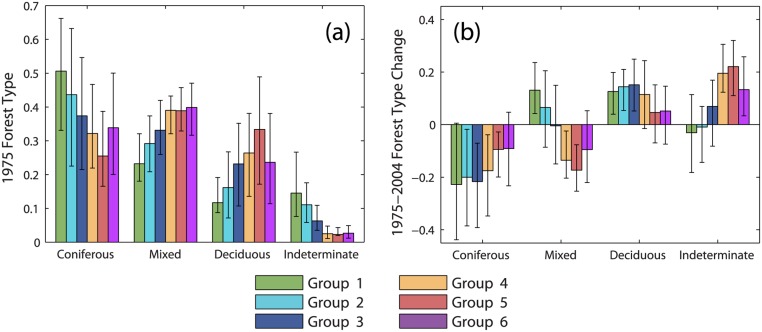
Initial landscape composition and changes in composition, 1975–2004. (a) Proportion of available forestland classified by 1975 forest type. (b) Change in forest type between 1975 and 2004, expressed as a proportion of forestland harvested prior to 2004 (negative values indicate loss; positive values indicate gain). Values were calculated for individual sample grid cells and then averaged within groups identified by cluster analysis of modeled cumulative harvest time series. Error bars represent the interquartile range.

The average initial composition of sample landscapes differed between groups, although there was a large amount of variability between landscapes in any single group (Fig 7a). In 1975, groups 1–2 contained more coniferous-dominant forest and less deciduous-dominant and mixed forest than other groups. Forest type classes were least balanced for group 1, with coniferous forest comprising 51% and deciduous forest 12% of available forestland. Conversely, group 5 contained more deciduous (33%) and less coniferous forest (25%) than other groups. The composition of groups 4 and 6 were very similar. The amount of forest of indeterminate type in 1975 was greatest for groups 1–2, a result of harvesting during the early 1970s.

Between 1975 and 2004, harvesting and subsequent forest recovery resulted in substantial changes in landscape composition (Fig 7b). On average all groups lost coniferous-dominant forestland. For groups 1–4, the coniferous forest lost amounted to about 20% of harvested forestland. For groups 1–3, much of this area transitioned to mixed or deciduous forest types, and the amount of forest classified as indeterminate remained little changed (recovery from early disturbance was balanced by disturbance in later years). Groups 4 and 5 lost both coniferous and mixed forest. This was partially balanced by an increase in deciduous forest for group 4, but a substantial proportion of total harvest area (~20%) was mapped as indeterminate due to high harvest rates during the 1990s and early 2000s.

## Discussion

During the spruce budworm outbreak of ca. 1972–1988, there were no legislative definitions or standards in place to regulate the practice of clearcutting. As the outbreak progressed, landowners engaged in extensive pre-salvage and salvage logging operations that typically took the form of large commercial clearcuts, much larger than would have been planned in the absence of the outbreak [20]. The FPA was designed to regulate the execution of clearcuts larger than 14 ha [53] (revised to 8 ha in 1999 [54]), and its implementation in 1991 marked a fundamental and abrupt change in forest policy and management.

Averaged across all grid cells, cumulative harvest area increased more or less linearly (Fig 2). From the cluster analysis, group 3 reflected this trend but contained only 20% of grid cells (Fig 3). The cumulative harvest time series of the other five groups differed substantially from the area-wide average. These groups of grid cells comprised segments of the study area with different management histories. Groups 1–2 (24% of grid cells) were differentiated from other groups by elevated rates of stand-replacing harvests during the budworm outbreak (Fig 4). Harvesting continued at moderated rates throughout the post-FPA period, then set against landscape conditions created by salvage logging. In contrast, harvesting within groups 4–6 (56% of grid cells) predominantly occurred during the post-FPA period. A large increase in group 5 harvest rates coincided with the end of the budworm outbreak and enactment of the FPA (Fig 3b). Group 4 harvest rates increased during the late 1980s, but most harvest area accrued post-FPA with particularly high partial harvest rates during the 1990s. Similarly, although group 6 harvest rates remained low to moderate post-FPA, most harvest area accrued during that time.

**Fig 8:**
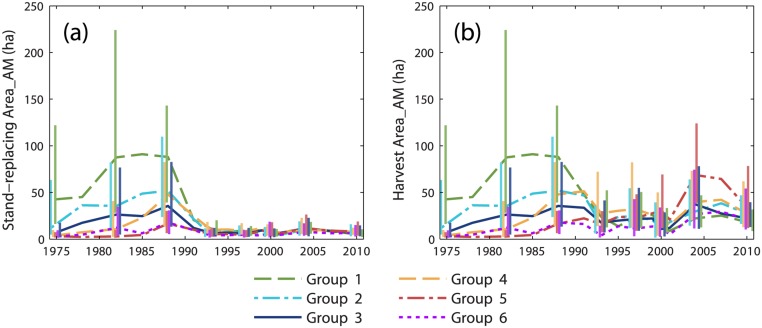
Changes in average harvest patch size through time. Area-weighted mean patch size for (a) the stand-replacing harvest class and (b) the combined stand-replacing and partial harvest class, calculated at each time series interval. Values were averaged within groups identified by cluster analysis of modeled cumulative harvest time series. Vertical bars represent the interquartile range. Bars are provided at a subset of dates and are offset horizontally to improve visual clarity.

Implementation of the FPA had the intended effect of reducing the size of clearcuts [25], and more generally the size of stand-replacing patches. Although our stand-replacing harvest class did not adhere to the FPA clearcut definition [36,37], average stand-replacing patch size calculated from individual harvest maps (rather than cumulative harvest maps) dropped dramatically between 1988 and 1991 for groups of grid cells affected by pre-FPA logging (Fig 8a). Average patch size of stand-replacing harvests varied dramatically between cells prior to 1991, often exceeding 100 ha for groups 1 and 2. By comparison, post-FPA stand-replacing patch sizes were uniformly low for all grid cells, with group averages below 15 ha. In contrast, overall harvest patch sizes (stand-replacing and partial harvest classes combined) remained relatively high post-FPA, with group 5 averages approaching the pre-FPA values of groups 1–2 (Fig 8b). The FPA placed a strong disincentive on clearcutting. State records indicate that clearcutting fell from 44% of annual harvest area in 1989 to <5% by 2000, and annual harvest area roughly doubled during that time [23,24]. Within typical post-FPA partial harvest blocks, timber is removed within and adjacent to machine trails, leaving a matrix of unharvested or lightly harvested area between trails. Partial harvests are composed of typically small areas of complete or nearly complete canopy removal intermixed with areas of light or negligible canopy disturbance.

Predominant patterns of cumulative landscape change created by pre- and post-FPA management regimes were revealed by time series of intact mature and regenerating forest metrics. Groups 1–2 most clearly represented salvage logging impact. During the 1970s and 1980s, salvage caused a rapid decrease in the average patch size of intact mature forest (Fig 5a) and a rapid increase in the average patch size of regenerating forest (particularly for group 1; Fig 6a). Intact mature forest patch density increased during this period (Fig 5b), but regenerating patch density changed relatively little (Fig 6b). Edge density between intact mature forest and regenerating forest increased (Figs 5c and 6c), but the average patch shape complexity of intact mature forest changed little, and the patch shape complexity of regenerating forest was comparable (Figs 5d and 6d). These trends were consistent with the subdivision of mature forest by salvage clearcut of large contiguous tracts of spruce-fir (e.g., Landsape A, Fig 2). Subsequent harvesting during the post-FPA period resulted in continued subdivision of intact mature forest at rates similar to the salvage period (Fig 5b). Otherwise, changes in configuration metrics of both intact mature forest and regenerating forest were considerably reduced. The primary effect of the post-FPA regime in grid cells with a prominent salvage logging legacy appears to have been the production of more small patches of intact mature forest, with little influence on other metrics.

Groups 4–6 represented segments of our study area that were little affected by salvage logging but heavily impacted by post-FPA harvesting. Similar to salvage in groups 1–2, over time there was substantial loss and subdivision of intact mature forest. In groups 4 and 5, average patch size decreased and patch density increased at rates that actually exceeded those of groups 1 and 2 pre-FPA (Fig 5a and 5b). Edge density and patch shape complexity increased sharply during the 1990s as well (Fig 5c and 5d). In group 6, cumulative harvest area was lower (Fig 3b) and the loss and subdivision of intact mature forest correspondingly reduced (Fig 5a and 5b), yet edge density and patch shape complexity increased to levels approaching or exceeding all other groups (Fig 5c and 5d). Post-FPA partial harvest practices resulted in complex patches of intact mature forest and high edge densities presumably due to residual inclusions of mature forest within harvest blocks. In sharp contrast to salvage logging in groups 1–2, average regenerating forest patch sizes in groups 5 and 6 remained very low (Fig 6a). Group 5 regenerating patch density increased rapidly in the 1990s and 2000s, surpassing all other groups by 2004 (Fig 6b). Group 5 edge density increased rapidly during the 2000s, ultimately exceeding the values of groups 1–2 at the end of the salvage period (Fig 6c) despite considerably less regenerating forest area (Fig 4a). Group 6 regenerating forest patch size, edge density, and shape complexity remained relatively low, but patch density steadily increased throughout the study period (Fig 6). Patterns within these groups indicate that post-FPA stand-replacing harvest patches were more numerous, much smaller, and simpler in shape compared to the pre-FPA salvage logging period (e.g., Landscape B, Fig 2).

Not surprisingly, groups that were most heavily impacted by budworm salvage logging were also those with the greatest amount of coniferous-dominant forestland in 1975 (groups 1–2, Fig 7a). Groups 4–6 contained less coniferous forestland and we attribute the contrast in management history and landscape change between groups 1–2 and groups 4–6 in large part to differences in initial landscape composition and vulnerability to the budworm outbreak. Initial composition set different segments of the study area on fundamentally different trajectories of landscape change. However, groups 4 and 6 differed very little in the relative abundance of 1975 forest types (Fig 7a), and comparisons between them suggest the influence of some other factor that affected post-FPA harvest patterns. Private ownership diversified greatly during the 1990s as industrial forest products companies restructured and sold their lands to investment entities, nonprofit conservation groups, high net-worth individuals, and other owner types [27,28]. We hypothesize that post-FPA differences in harvest rates, intensities, and trajectories of landscape change may have been influenced by differences in management incentives between different landowners (e.g., fiber production vs. resource conservation). Previous research documented differences in harvest rates between categories of ownership and ownership change [27], but the influence of owner-to-owner variability on patterns of landscape change remains unclear.

The relative importance of individual landowner behavior, public forest policy, and management or disturbance legacies on contemporary trajectories of landscape change is an important question with implications extending to regional forest planning, management, and conservation. Because multiple forest values are often maintained only when actions are integrated over large areas with diverse forest conditions, it is important to understand the relative influence of factors that act to either enhance or reduce landscape-scale heterogeneity. Within northern Maine, salvage logging introduced persistent heterogeneity at the scale of 5 km landscape units due to large clearcut operations. However, another important aspect of salvage legacy is loss of coniferous-dominant forest area (Fig 7b) and consequent homogenization of forest composition due to clearcut operations that failed to adequately regenerate spruce and fir [21]. Management under the FPA further homogenized landscape structure by effectively eliminating large clearcuts and incentivizing the expansion of partial harvesting. Under the post-FPA management regime, differences between landowners in management incentives, objectives, or strategies may provide an important source of landscape heterogeneity. Given the small amount of publicly owned forestland within the state of Maine (approximately 7% [55]), the sustainable management of Maine's forest resources will require a clearer understanding of landscape dynamics and management outcomes under various forms of private ownership, as well as closer consideration of the ways in which public policy may constrain outcomes.

The changes in landscape composition and configuration we have quantified imply potentially important impacts on forest ecology and wildlife. Salvage clearcuts created large blocks of early successional forest habitat, benefitting the federally threatened Canada lynx (*Lynx canadensis*) [56]. In Maine, the primary prey of lynx, snowshoe hare (*Lepus americanus*), are found at highest density within coniferous and mixed regenerating forest ~15–35 years post-harvest [57–59]. The current amount and configuration of this high-quality lynx foraging habitat is largely a product of pre-FPA salvage logging. Post-FPA harvest practices produce smaller and more numerous regenerating forest patches, promoting the subdivision of high-quality lynx foraging habitat [56]. Additionally, the large annual footprint of post-FPA partial harvesting and accelerated loss and subdivision of intact mature forest suggest rapid loss of suitable habitat or reduction of habitat quality for species that require features associated with mid- and late-successional forest, such as the American marten (*Martes americana*) [57]. For species that are either dependent upon landscape legacies or potentially impacted by rapid habitat modification, responses to contemporary management may be difficult to establish without knowledge of landscape history and disturbance trends. Our analysis demonstrated one approach by which landscape disturbance history can be defined and evaluated using a time series of Landsat-derived forest disturbance maps.

## Supporting Information

S1 Appendix Landsat image processing. Description of image processing steps performed prior to forest harvest and composition mapping.
(DOCX)

S2 Appendix Map validation. Description of the procedures used to validate cumulative forest harvest (1973–2010) and forest type maps (1975 and 2004), and a brief interpretation of validation outcomes.
(DOCX)
