# Supplementary material for: Evaluating the Impact of Abrupt Changes in Forest Policy and Management Practices on Landscape Dynamics: Analysis of a Landsat Image Time Series in the Atlantic Northern Forest
Source: PLoS One. 2015 Jun 24;10(6):e0130428. doi: 10.1371/journal.pone.0130428 (PMC4480355; doi:10.1371/journal.pone.0130428)
Supplement: S1 Appendix — Description of image processing steps performed prior to forest harvest and composition mapping. (DOCX) [file pone.0130428.s001.docx]

**S1 Appendix. Landsat image processing.** Description of image processing steps performed prior to forest harvest and composition mapping.

Forest harvest and composition maps were assembled from a time series of Landsat Multispectral Scanner (MSS), Thematic Mapper (TM), and Enhanced Thematic Mapper Plus (ETM+) images acquired during summer leaf-on conditions (Table 1). Consecutive images were spaced 1-4 years apart, as determined by the availability of high quality, predominantly cloud-free imagery. Images were either obtained from the U.S. Geological Survey (USGS) Earth Resources Observation and Science Center or available for use through other programs [1,2].

Change detection and composition mapping procedures were applied to forested pixels as identified by the 1993 Maine Gap Analysis Program (GAP) land cover map. The GAP map represents conditions near the midpoint of our time series, and discriminated forest from non-forest with an estimated 100% accuracy within our study area [2]. All images were geo-referenced to a previously rectified 1991 image that was used to produce the GAP map. TM and ETM+ images acquired 1988-2007 were rectified using a second-order polynomial transformation applied to 30-35 well distributed ground control points, with nearest neighbor resampling (RMSE <15 m). The 2010 TM image was obtained from the USGS with Level 1T Standard Terrain Correction and close inspection indicated that no further geocorrection was necessary. MSS images were rectified using a second-order polynomial transformation applied to 25-30 ground control points (RMSE <30 m) and resampled to 30 m by cubic convolution to match the spatial resolution of the TM/ETM+ imagery.

For each of the MSS and TM/ETM+ image sequences, a subset of image bands was selected for change detection and forest type mapping. TM/ETM+ red band 3, near-infrared band 4, and mid-infrared band 5 were retained, a combination that provides most of the image information content for northern temperate and boreal forests [3–5]. MSS green band 1, red band 2, and near-infrared band 4 were retained following the observation that near-infrared band 3 was less comparable to TM/ETM+ data [6]. Clouds and cloud shadows were delineated and masked using an on-screen digitization procedure. Cloud cover typically affected a small fraction of forestland (Table 1). Extensive cloud cover on 17 June 2007 was mitigated by substituting cloud-contaminated areas with TM image data acquired on 22 August 2007. The substitution of cloud-free data was not possible for images acquired in 1993 and 1997.

To facilitate visual interpretation, images were transformed to a common radiometric scale using a relative radiometric normalization procedure applied separately to MSS and TM/ETM+ imagery. A preliminary change detection procedure known as multivariate alteration detection was first applied to consecutive image pairs to identify pixels whose spectral characteristics had not changed [7]. Band values were extracted from a random sample of 5000 no-change pixels and linear normalization parameters were estimated using Theil-Sen regression [8]. Normalization parameters were used to derive a common radiometric scale for each band, preserving the full radiometric resolution of all images [9,10]. Normalization was performed to enhance visual consistency between images, and to reduce image-to-image differences in the impact of atmospheric effects on derived vegetation index values. However, the classification procedures used to produce forest harvest and composition maps (unsupervised classification guided by visual interpretation of Landsat images and ancillary data) do not assume a common radiometric scale across images. Normalization was therefore not a requirement [11], and normalization outcomes were accordingly evaluated by qualitative visual assessment only.

**References**

1. Lunetta RS, Lyon JG, Guindon B, Elvidge CD. North American Landscape Characterization Dataset Development and Data Fusion Issues. Photogramm Eng Remote Sensing. 1998;64:821–9.

2. Hepinstall JA, Sader SA, Krohn WB, Boone RB, Bartlett RI. Development and testing of a vegetation and land cover map of Maine. Orono, Maine, USA: Maine Agricultural and Forest Experiment Station, University of Maine; 1999. Technical Bulletin 173.

3. Horler DNH, Ahern FJ. Forestry information content of Thematic Mapper data. Int J Remote Sens. 1986;7:405–28.

4. Häme T. Spectral interpretation of changes in forest using satellite scanner images. Acta For Fenn. 1991;222:1–111.

5. Sader SA. Multispectral and seasonal characteristics of northern hardwood and boreal forest types in Maine. In: Elvidge C, editor. Image Processing ’89: Sparks, Nevada, 23 May 1989. Bethesda, Maryland, USA: The Society; 1990. p. 109–16.

6. Crist EP, Cicone RC. Comparisons of the dimensionality and features of simulated Landsat-4 MSS and TM data. Remote Sens Environ. 1984;14:235–46.

7. Canty MJ, Nielsen AA, Schmidt M. Automatic radiometric normalization of multitemporal satellite imagery. Remote Sens Environ. 2004;91:441–51.

8. Olthof I, Pouliot D, Fernandes R, Latifovic R. Landsat-7 ETM+ radiometric normalization comparison for northern mapping applications. Remote Sens Environ. 2005;95:388–98.

9. Du Y, Cihlar J, Beaubien J, Latifovic R. Radiometric normalization, compositing, and quality control for satellite high resolution image mosaics over large areas. IEEE Trans Geosci Remote Sens. 2001;39:623–34.

10. Du Y, Teillet PM, Cihlar J. Radiometric normalization of multitemporal high-resolution satellite images with quality control for land cover change detection. Remote Sens Environ. 2002;82:123–34.

11. Song C, Woodcock CE, Seto KC, Lenney MP, Macomber SA. Classification and change detection using Landsat TM data: When and how to correct atmospheric effects? Remote Sens Lett. 2001;75:230–44.
