# Supplementary material for: Evaluating the Impact of Abrupt Changes in Forest Policy and Management Practices on Landscape Dynamics: Analysis of a Landsat Image Time Series in the Atlantic Northern Forest
Source: PLoS One. 2015 Jun 24;10(6):e0130428. doi: 10.1371/journal.pone.0130428 (PMC4480355; doi:10.1371/journal.pone.0130428)
Supplement: S2 Appendix — Description of the procedures used to validate cumulative forest harvest (1973–2010) and forest type maps (1975 and 2004), and a brief interpretation of validation outcomes. (DOCX) [file pone.0130428.s002.docx]

**S2 Appendix. Map validation.** Description of the procedures used to validate cumulative forest harvest (1973-2010) and forest type maps (1975 and 2004), and a brief interpretation of validation outcomes.

**Validation overview**

The U.S. Forest Service Forest Inventory and Analysis (FIA) Program provides quality-assured measurements of forest attributes from a national network of field plots adhering to a statistically rigorous systematic sampling design [1,2]. We made extensive use of FIA data for validation of Landsat-derived forest harvest and type maps. Since 1999, 20% of FIA plots within Maine have been surveyed annually during 5-year inventory cycles. Earlier inventories were conducted at irregular intervals using different designs [2]. Under the modern inventory design, FIA plots consist of a center subplot with three satellite subplots, each 0.017 ha in size [2]. Subplots will generally fall within an area defined by a 3x3 pixel block (90x90 m), and subplots constitute 8% of that area. The suitability of FIA data for map validation is affected by mismatches in location and scale between FIA plots and pixel neighborhoods, uncertainty of field measurements, and for maps spanning long time periods, changes in inventory design. Validation using FIA data should be considered an assessment of agreement with an accepted and widely utilized source of information on forest conditions, rather than an assessment of accuracy against ground truth. The FIA program maintains the confidentiality of plot locations to protect the privacy of landowners and to preserve plot integrity [1,3]. True plot locations were made available for our use through a collaborative agreement with the USFS Northern Research Station FIA Program.

### Harvest time series validation procedure

Visual interpretation of TM/ETM+ imagery has been established as a credible source of reference data for the validation of forest disturbance maps [4–7]. Even very light partial harvests are discernible due to characteristic spectral response, texture, and clearly visible access roads. Stand-replacing and heavy partial harvests are similarly apparent in MSS imagery. However, visual discrimination of disturbance intensity classes is difficult and subjective. Forest inventory data provided by the FIA program provide a valuable alternative source of reference data. Forest age estimates at FIA plots represent the average age of overstory trees and, assuming they were established at the time of disturbance, the year of disturbance should be given by the year of field measurement minus stand age. However, if the overstory cohort was established from a seed source following disturbance or as advance regeneration prior to disturbance, the date of stand origin may be over- or underestimated, respectively. Field methods introduce additional uncertainty. Typically 2-3 trees are subjectively selected for aging. Age is estimated by coring and ring count at 1.37 m with the addition of a constant to approximate time elapsed between germination and growth to the height of coring [8]. Thomas et al. [9] validated forest disturbance time series (occurrence maps) by independent analyses of TM/ETM+ image interpretations and FIA forest age estimates. They concluded that validation is improved through the use of both reference sources, but they did not integrate the two into a single assessment. We found the visual discrimination of harvest intensity classes to be highly subjective, and FIA age to be an uncertain measure of time since disturbance. However, FIA plot data provide an objective basis for the discrimination of harvest intensity classes and image interpretation provides accurate identification of harvest dates. We adopted an approach to reference class labeling that leveraged the strength of one against the weakness of the other.

Our validation approach was based on the visual interpretation of satellite imagery over FIA plot locations to obtain reference class labels. Image interpretation was used to date harvest events; FIA plot data were used to discriminate stand-replacing and partial harvests. A trained image interpreter recorded the occurrence of a harvest provided spectral changes, image texture, and other contextual cues were consistent with harvesting in the vicinity of the plot and provided harvest operations appeared to have affected the majority of pixels within a 3x3 neighborhood surrounding plot center. Use of a 3x3 majority is consistent with FIA plot configuration [10]. A harvest recorded by visual interpretation was labeled stand-replacing provided FIA age dated stand origin to 1970 or later (allowing for advance regeneration established prior to 1973) and the field-assigned stand size class was either sapling or poletimber. A substantial fraction of plots sampled multiple forest condition classes, as identified by FIA field crews [8]. Age and stand size criteria were required of all sampled conditions. Age estimates were unreliable indicators of disturbance intensity for harvests that occurred after 1999, because age estimates frequently corresponded to a few remaining large stems rather than the new cohort which will eventually dominate the canopy. For plots harvested after 1999, an alternate approach was used to assign reference classes based on repeated plot measurements made during the 1999-2003 and 2004-2008 inventory cycles. Where the 5-year period between plot measurements included a harvest recorded by visual interpretation, that harvest was labeled stand-replacing if plot basal area (cross-sectional area of stems measured at 1.37 m) had been reduced by at least 70%. This basal area removal threshold was identified as that for which errors between stand-replacing and partial harvest classes were best balanced and mapped class extents least biased.

Of 671 FIA plots, we excluded 111 that contained non-forest cover types or field condition classes. We excluded 51 samples where a harvest was interpreted to have occurred after 1999 but the timing of plot measurements did not allow for both pre-harvest and post-harvest assessment of forest conditions. This occurred when the harvest either preceded plot measurement during the 1999-2003 inventory cycle or followed plot measurement during the 2004-2008 inventory cycle. The latter case included all harvests that occurred 2008-2010. The validation sample size of 509 was insufficient to produce reasonably precise estimates of class accuracy for individual time series intervals. We therefore aggregated intervals into the following six harvest validation classes: 1973-1988 stand-replacing harvest, 1988-1999 stand-replacing harvest, 1988-1999 partial harvest, 1999-2010 stand-replacing harvest, 1999-2010 partial harvest, and intact mature forest (no history of harvest, 1973-2010). Map and reference validation class labels for each sample were assigned in a manner consistent with the construction of cumulative harvest maps. Where multiple entries were mapped or interpreted to have occurred, the corresponding map or reference label was assigned based on the date of the first stand-replacing disturbance. If multiple partial harvests occurred, a validation class label of either 1988-1999 stand-replacing or 1999-2010 stand-replacing was assigned based on the date of the second entry. Reference labels were compared to the pixel locations coincident with plot centers, resulting in a per-pixel validation of harvest data.

Map and reference labels were compiled into an error matrix. Overall accuracy, user accuracy (the complement of class commission error), producer accuracy (the complement of class omission error), and corresponding standard error estimates were calculated using poststratified estimators [11,12]. Mapped pixel counts were calculated for each of the validation classes, and the validation sample was treated as a random sample stratified by validation class. Poststratification produces more efficient estimates of overall and producer accuracy than those obtained using formulae for a simple random sample; user accuracy estimates are equivalent [11,13]. Additionally, we evaluated the accuracy of our 2010 cumulative harvest map by further aggregating validation classes into regenerating, partially harvested, and intact mature forest.

### Forest type validation procedure

FIA plot measurements of coniferous and deciduous live tree basal area were used to derive reference class labels for validation of the 1975 and 2004 forest type maps. Reference labels were compared to pixel locations coincident with plot centers. The 2004 map was validated with data collected during the 1999-2003 inventory cycle. The 1975 map was validated using FIA data collected during the 1980-1982 inventory, which included a large proportion of samples consisting of a single 0.08 ha plot [14]. At these locations, multiple forest conditions within a 3x3 pixel neighborhood were less likely to have been sampled than under the modern plot design. To improve the comparability of validation data sets, we removed multiple-condition plots from the 2004 validation sample. Differences in dates between field data collection and satellite image acquisition resulted in cases where intervening harvests altered forest conditions. For 2004 map validation, we excluded samples where pixels within the 3x3 neighborhood surrounding plot center were mapped as harvested 1999-2004. For 1975 map validation, we excluded samples where neighborhood pixels were harvested 1975-1982. A total of 445 samples remained for validation of the 2004 map. Because accurate plot coordinates are known for only a small subset of plots sampled during the 1982 inventory, only 70 samples were available for validation of the 1975 map.

We identified coniferous-dominant and deciduous-dominant class thresholds for which errors were best balanced and mapped class extents least biased. To do so, we varied coniferous and deciduous threshold values from 50-95% in increments of 5%, assigned reference class labels based on threshold values, and calculated omission and commission error rates. We iteratively refined the maps and reevaluated error rates until a reasonable balance was achieved at the same threshold for both maps, facilitating meaningful comparisons of class extent between maps. Although we initially defined coniferous-dominant and deciduous-dominant classes using a 75% basal area threshold, we were better able to balance commission and omission error after adjusting class thresholds to 80% and 70%, respectively. An error matrix was compiled for each map based on these selected threshold values. Estimates of overall, user, and producer accuracy were calculated by poststratification [11,12].

**Interpretation of validation outcomes**

The overall agreement between map and reference harvest validation classes was high (Tables 2 and 3). The largest source of disagreement was confusion between harvest intensities, rather than confusion between harvest periods. Errors between stand-replacing and partial harvests were balanced for 1999-2010, indicating that the stand-replacing harvest class consistently represented harvests where >70% of basal area had been removed. Errors were similarly well balanced for 1973-1988, but not for 1988-1999. Use of field age and stand size criteria to infer stand-replacing disturbance may have been more appropriate for the clearcutting practices of the 1970s and 1980s than the partial harvest practices of the 1990s. Alternatively, harvest intensity may have been systematically under-represented for the 1990s, perhaps due to cloud cover in several 1990s images (Table 1). We adapted the three-date classification method to detect change in cloudy areas using preceding and succeeding images. As a consequence harvest intensity may have been under-estimated due to regrowth during the longer periods between clear observations. We cannot verify this due to insufficient validation sample sizes. Possible impacts to regenerating forest metrics are unknown, but the alternative of wholly missing harvests due to cloud cover would certainly have affected cumulative harvest area time series and intact mature forest metrics.

Forest type classes for both 1975 and 2004 were mapped with reasonably high overall accuracies (Tables 4 and 5). Overall accuracy and individual class accuracy estimates were higher for 1975 than for 2004. Although differences between FIA inventory designs and sample sizes complicate comparison, lower accuracies for the 2004 map probably reflect more heterogeneous forest landscape conditions. Off-diagonal entries in both error matrices indicated confusion between the mixed class and both coniferous- and deciduous-dominant classes. There was little confusion between coniferous and deciduous classes. Using coniferous-dominant and deciduous-dominant class thresholds of >80% and >70% basal area, respectively, errors were very well balanced for 2004 forest type classes and reasonably well balanced for 1975. User and producer accuracies for the 1975 map suggested under-representation of coniferous forest area and over-representation of mixed forest under these same class definitions, but the relatively small validation sample and correspondingly large standard error estimates made this inconclusive. Available validation data suggested that user and producer accuracies were best balanced under these class definitions. Note that had more historic field plot locations been available, perhaps we could have balanced errors using the original class thresholds of 75%. Nonetheless, our validation procedure served the purpose of ensuring that forest type classes in both maps represented the same forest conditions, so that map comparisons were meaningful.

**References**

1. Smith WB. Forest inventory and analysis: a national inventory and monitoring program. Environ Pollut. 2002;116 Suppl:S233–42.

2. McRoberts RE, Bechtold WA, Patterson PL, Scott CT, Reams GA. The Enhanced Forest Inventory and Analysis Program of the USDA Forest Service: historical perspective and announcement of statistical documentation. J For. 2005;103:304–8.

3. Coulston JW, Riitters KH, McRoberts RE, Reams GA, Smith WD. True versus perturbed forest inventory plot locations for modeling: a simulation study. Can J For Res. 2006;36:801–7.

4. Cohen WB, Fiorella M, Gray J, Helmer E, Anderson K. An efficient and accurate method for mapping forest clearcuts in the Pacific Northwest using Landsat imagery. Photogramm Eng Remote Sensing. 1998;64:293–300.

5. Sader SA, Bertrand M, Wilson EH. Satellite change detection of forest harvest patterns on an industrial forest landscape. For Sci. 2003;49:341–53.

6. Sader SA, Legaard KR. Inclusion of forest harvest legacies, forest type, and regeneration spatial patterns in updated forest maps: A comparison of mapping results. For Ecol Manage. 2008;255:3846–56.

7. Cohen WB, Yang Z, Kennedy R. Detecting trends in forest disturbance and recovery using yearly Landsat time series: 2. TimeSync — Tools for calibration and validation. Remote Sens Environ. 2010;114:2911–24.

8. U.S. Forest Service. Forest inventory and analysis national core field guide, volume1: field data collection procedures for phase 2 plots, Version 6.0. Washington DC, USA: US Department of Agriculture, Forest Service, Forest Inventory and Analysis; 2012.

9. Thomas NE, Huang C, Goward SN, Powell S, Rishmawi K, Schleeweis K, et al. Validation of North American Forest Disturbance dynamics derived from Landsat time series stacks. Remote Sens Environ. 2011;115:19–32.

10. Cooke WH. Forest/non-forest stratification in Georgia with Landsat Thematic Mapper data. In: McRoberts RE, Reams GA, Van Deusen PC, editors. Proceedings of the First Annual Forest Inventory and Analysis Sympossium. St. Paul: US Forest Service, North Central Research Station; 2000. p. 28–30.

11. Card DH. Using known map category marginal frequencies to improve estimates of thematic map accuracy. Photogramm Eng Remote Sensing. 1982;48:431–9.

12. Zhu Z, Yang L, Stehman SV, Czaplewski RL. Accuracy assessment for the U.S. Geological Survey regional land-cover mapping program: New York and New Jersey Region. Photogramm Eng Remote Sensing. 2000;66:1425–35.

13. Stehman S V. Model-assisted estimation as a unifying framework for estimating the area of land cover and land-cover change from remote sensing. Remote Sens Environ. 2009;113:2455–62.

14. U.S. Forest Service. Resources evaluation field instructions for Maine: 1980-1981. Broomall, Pennsylvania, USA: US Department of Agriculture, Forest Service, Northeastern Experiment Station, Renewable Resources Evalutation Work Unit; 1981.
